# Asymmetric Nerve Enlargement: A Characteristic of Leprosy Neuropathy Demonstrated by Ultrasonography

**DOI:** 10.1371/journal.pntd.0004276

**Published:** 2015-12-08

**Authors:** Helena Barbosa Lugão, Marcello Henrique Nogueira-Barbosa, Wilson Marques Jr., Norma Tiraboschi Foss, Marco Andrey Cipriani Frade

**Affiliations:** 1 Dermatology Division, Department of Internal Medicine, Ribeirão Preto Medical School, University of São Paulo, Ribeirão Preto, São Paulo, Brazil; 2 Radiology Division, Department of Internal Medicine, Ribeirão Preto Medical School, University of São Paulo Ribeirão Preto, São Paulo, Brazil; 3 Department of Neurology, Ribeirão Preto Medical School, University of São Paulo, Ribeirão Preto, São Paulo, Brazil; Hospital Infantil de Mexico Federico Gomez, UNITED STATES

## Abstract

**Background:**

Neurological involvement occurs throughout the leprosy clinical spectrum and is responsible for the most feared consequences of the disease. Ultrasonography (US) provides objective measurements of nerve thickening and asymmetry. We examined leprosy patients before beginning multi-drug therapy aiming to describe differences in US measurements between classification groups and between patients with and without reactions.

**Methodology/Principal Findings:**

Eleven paucibacillary (PB) and 85 multibacillary (MB) patients underwent nerve US. Twenty-seven patients had leprosy reactions (type 1, type 2 and/or acute neuritis) prior to US. The ulnar (at the cubital tunnel–Ut–and proximal to the tunnel–Upt), median (M) and common fibular (CF) nerves were scanned to measure cross-sectional areas (CSAs) in mm^2^ and to calculate the asymmetry indexes ΔCSA (absolute difference between right and left CSAs) and ΔUtpt (absolute difference between Upt and Ut CSAs). MB patients showed greater (p<0.05) CSAs than PB at Ut (13.88±11.4/9.53±6.14) and M (10.41±5.4/6.36±0.84). ΔCSAs and ΔUtpt were similar between PB and MB. The CSAs, ΔCSAs and ΔUtpt were similar between PB patients with reactions compared to PB patients without reactions. MB patients with reactions showed significantly greater CSAs (Upt, Ut and M), ΔCSAs (Upt and Ut) and ΔUtpt compared to MB patients without reactions. PB and MB showed similar frequencies of abnormal US measurements. Patients with reactions had higher frequency of nerve thickening and similar frequency of asymmetry to those without reactions.

**Conclusions/Significance:**

This is the first study to investigate differences in nerve involvement among leprosy classification groups using US before treatment. The magnitude of thickening was greater in MB and in patients with reactions. Asymmetry indexes were greater in patients with reactions and did not significantly differ between PB and MB, demonstrating that asymmetry is a characteristic of leprosy neuropathy regardless of its classification.

## Author Summary

Leprosy is an infectious disease that affects the peripheral nerves, leading to nerve thickening, asymmetry and dysfunction; therefore, early detection of leprosy neuropathy is important for preventing deformities and disabilities. We examined peripheral nerve involvement using ultrasonography (US) in 96 leprosy patients prior to treatment, aiming to better understand differences in neuropathy patterns between leprosy classification groups and between patients with and without leprosy reactions. Patients underwent bilateral US of the ulnar (at the cubital tunnel and proximal to the tunnel), median and common fibular nerves to measure thickening and asymmetry. We found that nerve thickening was more severe in patients with a high bacillary load (multibacillary) and in those with previous reactions. Nerve asymmetry measurements were greater in the patients with previous reactions. Asymmetry did not differ significantly between the paucibacillary and multibacillary patients, demonstrating that asymmetry is a characteristic of leprosy neuropathy regardless of its classification.

## Introduction

Neurological involvement is present throughout the leprosy clinical spectrum, and nerve impairment is responsible for the most feared consequences of the disease; therefore, some authors advocate that leprosy should be regarded as a chronic neurological condition rather than a skin disease [1–5]. Several authors have postulated that tuberculoid patients have asymmetric nerve thickening, lepromatous patients have symmetric and diffuse involvement, and borderline patients have variable and usually intense nerve enlargement [1,3,6,7]. Leprosy reactions (acute neuritis, types 1 and 2 reactions) can lead to additional nerve damage due to immune-mediated mechanisms. They may be superimposed on the chronic course of the disease and require immediate treatment [1,3,5,6].

Neurophysiologic and imaging studies can be used to investigate neurological impairment in leprosy. Although neurophysiology provides detailed information about dysfunction of affected nerves, it does not reveal anatomic changes, such as thickening and fascicular pattern changes [8,9]. High-resolution ultrasonography (US) permits examination of multiple nerve trunks over a long course in a few minutes, and compared with magnetic resonance imaging, US is considered more accessible and reasonably precise [8,10–12]. Furthermore, it is reported that US is more accurate than clinical palpation for assessment of peripheral nerve enlargement [11] and it provides objective measurements of peripheral nerve thickening and asymmetry [13]. To our knowledge, no published studies have investigated differences in US nerve measurements across the leprosy spectrum.

The purpose of this study was to investigate peripheral nerve thickening and asymmetry, as evaluated through US measurements, in leprosy patients, examining differences between leprosy types grouped according to the Ridley-Jopling and WHO classifications and to assess the influence of leprosy reactions on US findings.

## Materials and Methods

### Ethics statement

The Ethics Committee of the Clinics Hospital of Ribeirão Preto Medical School approved the study (process n°02663112.0.0000.5440). Written informed consent was obtained from all participants. Some participants were minors and their parents provided written consent on behalf of them.

### Subjects

Ninety-six consecutive leprosy patients that were referred to Leprosy Reference Center at the Clinics Hospital of Ribeirão Preto Medical School were included in the study and underwent bilateral high-resolution US of the peripheral nerves before starting World Health Organization (WHO) multi-drug therapy. Leprosy diagnosis was established based on clinical signs and symptoms, skin smears, skin biopsy, and neurophysiologic examination when necessary. Patients were classified according to the Ridley-Jopling [14] classification in five groups: tuberculoid (TT), borderline-tuberculoid (BT), borderline-borderline (BB), borderline-lepromatous (BL), and lepromatous (LL). Patients were also grouped in paucibacillary (PB) and multibacillary (MB) according to the WHO operational classification [15].

Medical charts were reviewed for the identification of any leprosy reactions prior to US evaluation. Leprosy reactions were classified as cutaneous reactions (type 1 or 2) associated or not with acute neuritis. Type 1 cutaneous reactions were defined as presence of erythema and edema of skin lesions. There may be accompanying neuritis and edema of the hands, feet, and face. Type 2 reactions (erythema nodosum leprosum) were defined as presence of tender subcutaneous skin lesions. There may be accompanying neuritis, iritis, arthritis, orchitis, dactylitis, lymphadenopathy, edema, and fever. Neuritis was diagnosed if patients presented acute inflammation of one or more peripheral nerve trunk detected by swelling and/or functional impairment with spontaneous nerve pain and/or nerve tenderness on palpation. To statistical analysis patients were divided according to the presence of any kind of reaction prior to US examination or absence of reactions.

Patients with a history of diabetes mellitus, hypothyroidism, human immunodeficiency virus infection, trauma-related peripheral nerve disease or alcoholism were excluded from the study.

### Ultrasonography

Musculoskeletal radiologists with previous fellowship training in nerve imaging performed all US sessions using a 12-MHz linear transducer model HDI-11 (Philips Medical Systems, Bothell, Washington, USA). Patients were examined in a seated position with 45° flexed elbows and 90° flexed knees. The ulnar (at the cubital tunnel area–Ut–and proximal to the tunnel–Upt), median (M) and common fibular (CF) nerves were systematically scanned along the transverse and longitudinal axes. Ulnar nerves were scanned from the middle third of the arm to the middle third of the forearm. M nerves were evaluated at the middle and distal thirds of the forearm. CF nerves were evaluated from the distal third of the thigh to the knee at the fibular head. In some cases, it was not possible to examine nerves bilaterally due to amputation, cutaneous ulcers or other cutaneous alterations at the site of examination.

Nerve cross-sectional areas (CSAs) were measured by freehand delimitation at the inner borders of the echogenic rims of the nerves. The measurements were performed using the electronic cursor at the time of examination, and the CSAs were assessed at the level of maximum nerve thickening. Ulnar nerve maximum CSAs were measured in two different regions: above the medial epicondyle proximal to the cubital tunnel (Upt) and at the cubital tunnel (Ut).

CSA measurements were used to calculate nerve asymmetry as follows: (1) the differential CSA index (ΔCSA), which was calculated as the absolute difference between CSAs for each nerve point from one side to the contralateral side, and (2) the differential Ut-Upt index (ΔUtpt) of the ulnar nerves, which was calculated as the absolute difference between the largest and smallest CSAs of Upt and Ut points of ulnar nerves on the same side. High ΔCSA values reflect nerve asymmetry with the contralateral nerve. High ΔUtpt values reflect non-uniform and focal thickening of the ulnar nerve between the tunnel and pre-tunnel areas.

For the classifications of the CSA, ΔCSA and ΔUtpt values as normal or abnormal, we used previously published values obtained from healthy volunteers [13], considering as abnormal values that were greater than the mean plus 2 standard deviations. Additionally, to compare the PB and MB groups, we performed two different analyses: one including all patients and another including only those patients with at least one abnormal measurement for each variable (CSA, ΔCSA and ΔUtpt). With second analysis, we expected to reduce the possible bias due to the existence of different frequencies of abnormalities in PB and MB.

### Statistical analysis

Statistical analysis was performed using JMP software version 10.0 (SAS Institute Inc., Cary, NC). We performed the Wilcoxon test to compare the means of two groups. To analyze differences between the means of three or more groups, we performed the Kruskal-Wallis test. For the comparison of proportions, we used the two-tailed Fisher’s exact test. Probability (p) values less than 0.05 were considered significant.

## Results

The average age ± standard deviation of the patients was 45.9 ± 16 years (age range 16–85 years); 56 (58.3%) of the patients were men, and 40 (41.7%) were women. The clinical classifications and incidences of neuritis, type 1 and/or type 2 cutaneous reactions occurring prior to US are presented in Table 1. For clinical data and CSAs values of each patient see supporting information (S1 Table).

**Table 1:** Clinical data for the patients included in the study.

Leprosy classification				Leprosy reactions			
WHO*	n (%)	RJ**	n (%)	Neuritis	Type 1	Type 2	No previous reactions
PB	11 (11.5%)	TT	11 (11.5%)	2	1	0	8
MB	85 (88.5%)	BT	31 (32.3%)	3	2	0	26
		BB	33 (34.4%)	2	4	0	28
		BL	13 (13.5%)	3	9	3	2
		LL	8 (8.3%)	2	0	3	5
Total	96		96	12	16	6	69

n: number of patients, with percentages in parentheses.

*WHO: Operational classification proposed by the World Health Organization.

** RJ: Ridley-Jopling classification.

Table 2 shows the results for CSA, ΔCSA and ΔUtpt of the nerves studied for the five types of leprosy according to the Ridley-Jopling classification.

**Table 2:** CSAs, ΔCSAs and ΔUtpt results. Patients were classified according to the Ridley-Jopling classification.

			Group					
			TT	BT	BB	BL	LL	p-value
Variable	Nerve		11 patients	31 patients	33 patients	13 patients	8 patients	
CSA (mm^2^)	**Ulnar (Upt)**	n	22	58	66	26	16	
		mean±SD	7±2.1	8.9±8.5	9±7	11.3±6.1	14.9±9.1	0.0006*
		95% CI	[6.1–7.9]	[6.7–11.1]	[7.3–10.8]	[8.8–13.8]	[10.1–19.7]	
		abnormal	3 (13.6%)	9 (15.5%)	16 (24.4%)	13 (50%)	10 (62.5%)	
	**Ulnar (Ut)**	n	22	60	66	26	16	
		mean±SD	8.6±5.5	12.4±14.9	10.8±6.2	10.6±4.7	15.6±4.6	<0.0001*
		95% CI	[6.1–11]	[8.6–16.3]	[9.3–12.3]	[8.7–12.5]	[13.1–18]	
		abnormal	4 (18.2%)	14 (23.3%)	24 (36.4%)	11 (42.3%)	13 (81.3%)	
	**Median**	n	20	61	63	26	16	
		mean±SD	6.2±1	8±4.5	8.4±4.4	11.6±6.2	12±4.8	<0.0001*
		95% CI	[5.7–6.7]	[6.9–9.2]	[7.3–9.5]	[9.1–14.1]	[9.5–14.6]	
		abnormal	0 (0%)	17 (27.9%)	18 (28.6%)	10 (38.5%)	9 (56.3%)	
	**Common Fibular**	n	22	62	64	26	16	
		mean±SD	16.8±13.1	16.4±9.3	16.1±8.6	21.1±10.2	20.3±7.6	0.0049*
		95% CI	[11–22.6]	[14–18.7]	[14–18.6]	[17–25.2]	[16.3–24.4]	
		abnormal	6 (27.3%)	19 (30.6%)	17 (26.6%)	12 (46.2%)	9 (56.3%)	
ΔCSA (mm^2^)	**Ulnar (Upt)**	n	11	28	33	13	8	
		mean±SD	1.7±1.9	5.1±11.2	3.2±4.5	2.6±3.2	7.2±8.7	0.5027
		95% CI	[0.4–2.9]	[0.7–9.4]	[1.6–4.7]	[0.7–4.5]	[0–14.5]	
		abnormal	3 (27.3%)	8 (28.6%)	9 (27.3%)	4 (30.8%)	5 (62.5%)	
	**Ulnar (Ut)**	n	11	29	33	13	8	
		mean±SD	3.4±4.7	6.0±17.9	4.2±5.9	2.8±2.6	4.1±2.4	0.3665
		95% CI	[0.2–6.6]	[0–12.7]	[2.1–6.3]	[1.2–4.4]	[2.1–6.0]	
		abnormal	3 (27.3%)	8 (27.6%)	16 (48.5%)	5 (38.5%)	6 (75%)	
	**Median**	n	10	30	31	13	8	
		mean±SD	0.8±0.6	1.6±2.5	2.4±4.1	1.9±2.4	1.8±1.9	0.7688
		95% CI	[0.4–1.2]	[0.7–2.5]	[0.9–3.9]	[0.4–3.3]	[0.2–3.3]	
		abnormal	0 (0%)	5 (16.7%)	6 (19.4%)	1 (7.7%)	3 (37.5%)	
	**Common Fibular**	n	11	31	32	13	8	
		mean±SD	7.3±15.6	4.5±7.1	3.2±3.9	7.1±8.8	2.2±2.1	0.6314
		95% CI	[0–17.7]	[1.9–7.2]	[1.8–4.6]	[1.7–12.4]	[0.4–4.0]	
		abnormal	3 (27.3%)	11 (35.5%)	11 (34.4%)	7 (53.9%)	2 (25%)	
ΔUtpt (mm^2^)	**Ulnar (Ut and Upt)**	n	22	57	66	26	16	
		mean±SD	3.4±4.9	5.0±8.0	3.4±4.1	3.9±4.0	7.3±6.0	0.0238*
		95% CI	[1.2–5.5]	[2.9–7.1]	[2.4–4.4]	[2.3–5.5]	[4.1–10.5]	
		abnormal	7 (31.8%)	13 (22.8%)	16 (24.2%)	7 (26.9%)	11 (68.8%)	

n: number of nerves; SD: standard deviation; 95% CI: 95% confidence interval; abnormal: number of nerves with abnormal measurements and percentages in parenthesis (values were considered abnormal when they exceeded the mean + 2SD value obtained from healthy volunteers).

p-value by Kruskal-Wallis test.

* Statistically significant.

For all nerve points studied, we observed maximum mean values of CSA in LL and BL patients; the percentages of enlarged nerves (abnormal CSAs) were also greater in these two groups, reaching 81.3% incidence of enlarged ulnar nerves at the cubital tunnel (Ut) in LL patients. Although the mean values of ΔCSAs showed variable results, with maximum values in different types of leprosy, we observed higher incidences of asymmetric nerves (abnormal ΔCSAs) in LL and BL patients. LL patients showed the maximum ΔUtpt mean and the highest percentage of nerve asymmetry considering this measurement.

Table 3 shows the CSAs, ΔCSAs and ΔUtpt according to the WHO operational classification for all patients and for the patients with at least one abnormal measurement for each variable. The two analyses yielded similar results, with the MB patients displaying greater CSAs, ΔCSAs, ΔUtpt and frequency of abnormalities at the Upt, Ut and M nerves. On the other hand, at the CF nerve, the PB patients showed similar or slightly greater CSAs and ΔCSAs.

**Table 3:** CSAs, ΔCSAs and ΔUtpt results for all patients and for those with at least one abnormal measurement for each variable. Patients were classified according to the WHO operational classification.

		All patients					Patients with at least one abnormal measurement				
Variable	Nerve	PB		MB		p-value	PB		MB		p-value
CSA (mm^2^)	**Ulnar (Upt)**	n = 22		n = 166			n = 16		n = 114		
		mean±SD	6.99±2.06	mean±SD	9.9±7.78	0.1110	mean±SD	7.64±1.91	mean±SD	11.64±8.79	0.0729
		95% CI	[6.07–7.9]	95% CI	[8.71–11.09]		95% CI	[6.63–8.66]	95% CI	[10–13.27]	
		abnormal	3 (13.6%)	abnormal	48 (28.9%)		abnormal	3 (18.8%)	abnormal	48 (42.1%)	
	**Ulnar (Ut)**	n = 22		n = 168			n = 16		n = 116		
		mean±SD	8.56±5.48	mean±SD	11.8±9.99	0.0038*	mean±SD	9.53±6.14	mean±SD	13.88±11.4	0.0061*
		95% CI	[6.14–10.99]	95% CI	[10.28–13.32]		95% CI	[6.26–12.8]	95% CI	[11.78–15.97]	
		abnormal	4 (18.2%)	abnormal	65 (38.7%)		abnormal	4 (25%)	abnormal	62 (53.5%)	
	**Median**	n = 20		n = 166			n = 14		n = 116		
		mean±SD	6.18±1.03	mean±SD	9.12±4.97	0.0015*	mean±SD	6.36±0.84	mean±SD	10.41±5.4	0.0002*
		95% CI	[5.7–6.66]	95% CI	[8.36–9.88]		95% CI	[5.87–6.84]	95% CI	[9.41–11.4]	
		abnormal	0 (0%)	abnormal	54 (32.5%)		abnormal	0 (0%)	abnormal	54 (46.6%)	
	**Common Fibular**	n = 22		n = 168			n = 16		n = 118		
		mean±SD	16.76±13.09	mean±SD	17.38±9.18	0.1729	mean±SD	18.64±14.98	mean±SD	19.82±9.76	0.1911
		95% CI	[10.96–22.56]	95% CI	[15.99–18.78]		95% CI	[10.66–26.63]	95% CI	[18.04–21.6]	
		abnormal	6 (27.3%)	abnormal	57 (33.9%)		abnormal	6 (37.5%)	abnormal	57 (48.3%)	
ΔCSA (mm^2^)	**Ulnar (Upt)**	n = 11		n = 82			n = 8		n = 57		
		mean±SD	1.65±1.93	mean±SD	4.12±7.74	0.2811	mean±SD	2.01±2.12	mean±SD	5.58±8.9	0.2267
		95% CI	[0.35–2.94]	95% CI	[2.42–5.82]		95% CI	[0.24–3.79]	95% CI	[3.22–7.95]	
		abnormal	3 (27.3%)	abnormal	26 (31.7%)		abnormal	3 (37.5%)	abnormal	26 (45.6%)	
	**Ulnar (Ut)**	n = 11		n = 83			n = 8		n = 57		
		mean±SD	3.4±4.73	mean±SD	4.58±11.19	0.5560	mean±SD	4.41±5.25	mean±SD	6.3±13.17	0.4720
		95% CI	[0.22–6.58]	95% CI	[2.14–7.02]		95% CI	[0.02–8.8]	95% CI	[2.8–9.79]	
		abnormal	3 (27.3%)	abnormal	35 (42.2%)		abnormal	3 (37.5%)	abnormal	35 (61.4%)	
	**Median**	n = 10		n = 82			n = 7		n = 57		
		mean±SD	0.79±0.55	mean±SD	1.97±3.11	0.3228	mean±SD	0.86±0.48	mean±SD	2.5±3.58	0.2181
		95% CI	[0.4–1.18]	95% CI	[1.28–2.65]		95% CI	[0.41–1.3]	95% CI	[1.55–3.45]	
		abnormal	0 (0%)	abnormal	15 (18.3%)		abnormal	0 (0%)	abnormal	15 (26.3%)	
	**Common Fibular**	n = 11		n = 84			n = 8		n = 59		
		mean±SD	7.25±15.62	mean±SD	4.21±6.13	0.5256	mean±SD	9.39±18.14	mean±SD	5.47±6.94	0.5167
		95% CI	[0–17.74]	95% CI	[2.88–5.54]		95% CI	[0–24.55]	95% CI	[3.66–7.27]	
		abnormal	3 (27.3%)	abnormal	31 (36.9%)		abnormal	3 (37.5%)	abnormal	31 (52.5%)	
ΔUtpt (mm^2^)	**Ulnar (Ut and Upt)**	n = 22		n = 165			n = 10		n = 65		
		mean±SD	3.35±4.87	mean±SD	4.41±5.95	0.1728	mean±SD	6.02±6.34	mean±SD	8.65±7.66	0.1652
		95% CI	[1.19–5.51]	95% CI	[3.49–5.32]		95% CI	[1.48–10.56]	95% CI	[6.74–10.54]	
		abnormal	7 (31.8%)	abnormal	47 (28.5%)		abnormal	7 (70%)	abnormal	47 (72.3%)	

n: number of nerves; SD: standard deviation; 95% CI: 95% confidence interval; abnormal: number of nerves with abnormal measurements and percentages in parenthesis (values were considered abnormal when they exceeded the mean + 2SD value obtained from healthy volunteers).

p-values were determined using the Wilcoxon test.

* Statistically significant.

Because borderline patients show variable nerve impairment, we compared only the two polar forms, thereby aiming to better characterize and highlight nerve thickening and asymmetry. For this analysis, we selected only TT (n = 8) and LL (n = 8) patients who had at least one abnormal measurement for each variable. Significantly greater CSAs were observed in the LL patients at the Upt (7.64±1.91 /14.9±9.08 mm^2^), Ut (9.53±6.14 / 15.56±4.57 mm^2^) and M (6.36±0.84 / 12.04±4.75 mm^2^) nerves; no significant difference was observed at the CF nerve. Although the ΔCSAs were also greater in LL patients, no statistically significant differences were observed at the Upt (2.01±2.12 / 8.11±8.96 mm²), Ut (4.41±5.25 / 4.44±2.23 mm^2^) and M nerves (0.86±0.48 / 1.94±1.91 mm^2^). At the CF nerve, the TT patients showed a non-significantly greater ΔCSA (9.29±18.14 / 2.4±2.23 mm^2^). It is important to report that one TT patient had the maximum ΔCSA value observed in the study at this nerve (52.7mm^2^). No significant difference in the ΔUtpt was observed between TT and LL (6.02±6.34 / 8.18±5.93 mm^2^).

The CSAs, ΔCSAs and ΔUtpt were similar between PB patients with previous leprosy reactions (neuritis, type 1 and/or type 2 reactions) compared to PB patients with no reaction. MB patients with reactions showed significantly greater CSAs compared to MB patients without reactions at the Upt (14.20±11.09 / 8.20±5.12 mm^2^), Ut (14.73±15.73 / 10.62±6.13 mm^2^) and M nerves (10.88±5.99 / 8.40±4.32 mm^2^). ΔCSAs were also significantly greater in MB patients with reactions at the Upt (7.39±11.07 / 2.84±5.59 mm^2^) and Ut nerves (8.33±19.29 / 3.06±4.61 mm^2^). In addition, the ΔUtpt was significantly greater in MB patients with previous leprosy reactions (5.82±7.36 / 3.85±5.21 mm^2^).

Considering that abnormalities in at least one US measurement reflect neuropathy, we sought to evaluate differences in the frequencies of abnormalities between PB and MB patients and also between patients who did and did not have reactions prior to US. The results of this analysis are presented in Table 4.

**Table 4:** Percentage of patients with at least one abnormal CSA, ΔCSA or ΔUtpt measurement and those with all nerve measurements within the range of normality grouped according to the WHO operational classification and according to the presence or absence of reactions.

		PB	MB	p-value	No reaction	Previous reactions	p-value
CSA	At least one abnormal measurement	72.7%	69.4%	1.000	63.8%	85.2%	0.0409*
	Normal	27.3%	30.6%		36.2%	14.8%	
ΔCSA	At least one abnormal measurement	72.7%	69.4%	1.000	65.2%	81.5%	0.1433
	Normal	27.3%	30.6%		34.8%	18.5%	
ΔUtpt	At least one abnormal measurement	45.5%	40%	0.7534	36.8%	51.8%	0.2476
	Normal	54.5%	60%		63.2%	48.2%	

Values were considered abnormal when they exceeded the mean + 2SD value obtained from healthy volunteers.

p-value by two-tailed Fisher’s test.

* Statistically significant.

## Discussion

We found that nerve asymmetry detected on US is characteristic of leprosy, with similar frequencies of abnormal measurements found in PB and MB patients. Furthermore, we observed a tendency toward higher ΔCSA and ΔUtpt values in the latter group. As expected, we also found that thickening (CSA) of the peripheral nerves was more pronounced in MB. Another important finding was that MB patients with previous leprosy reactions (neuritis, type 1 and/or type 2) had greater CSA, ΔCSA and ΔUtpt values than MB patients without reactions; however, among the PB patients there was no significant difference in nerve thickening and asymmetry comparing the groups with and without reactions.

This is the first study in which differences in peripheral nerve thickening and asymmetry among patients of different leprosy classification groups were systematically investigated using accurate US measurements prior to specific treatment (WHO multi-drug therapy); the inclusion of patients at different stages of treatment in previous studies [10,11,16] could weaken conclusions about variations in the pattern of nerve involvement. Two studies in which US evaluation was performed before treatment have been reported, but those studies did not address differences between leprosy classification types [8,13].

There is a growing interest in US as a diagnostic tool for peripheral neuropathies. Nerve palpation is subjective and requires expertise [5], and even among trained professionals, the reliability of palpation of superficial peripheral nerves is unsatisfactory, with poor agreement between evaluators [17]. In a study that compared clinical examination and US of peripheral nerves in 20 leprosy patients and 30 healthy volunteers, Jain et al. [11] concluded that clinical examination was subjective and inaccurate, whereas US provided an objective evaluation of nerve damage and could identify more extensive involvement. Another previous study showed that US abnormalities may be present in patients with normal neurophysiological findings [8]. The concept that US should always be performed in addition to neurophysiological studies during the investigation of peripheral neuropathies is currently gaining strength [8,9,12].

In our study, we found that peripheral nerve involvement, objectively evaluated by US, is common in all types of leprosy. Considering that the presence of one or more abnormal nerve measurements reflects neuropathy, we observed similar frequencies of neuropathy in the PB and MB patients. The frequency of abnormalities was high even among the PB patients (72.7% of the PB patients had at least one thickened or asymmetric nerve), suggesting that enlargement and asymmetry of the peripheral nerves may be more frequently detected on US, corroborating the findings of Jain et al. [11]. These results support the idea that leprosy is a neurological disease [1–3,5] and reinforce the importance of conducting a detailed neurological exam for all patients.

The ulnar nerve was the most commonly involved nerve in MB patients; up to 81.3% of LL patients showed abnormal CSA values in the cubital tunnel area. Furthermore, thickening of peripheral nerves was also frequent in PB patients, especially at the common fibular nerve. The thickening was clearly more pronounced in MB patients at superior limb nerves, even when we analyzed only the group of patients in which some neuropathy was detected at US: the CSAs were significantly greater in the MB group, and the 95% confidence interval showed no overlap between PB and MB mean values at the ulnar (Upt and Ut) and median nerves. At the common fibular nerve, no difference was found between PB and MB patients; furthermore, the common fibular nerve was the most frequently affected nerve in PB patients, in agreement with observations from clinical studies that suggest that this nerve can be impaired even early in the disease course [7,18].

Clinical expertise and previous studies indicate that PB patients (especially TT) have asymmetric nerve enlargement, whereas MB patients, notably the patients exhibiting the lepromatous polar form of the disease, have symmetric and diffuse involvement [1,6]. Frade et al. [13] have shown that ΔCSA and ΔUtpt possess high specificity for a diagnosis of leprosy when leprosy patients are compared to healthy individuals and that these measurements allow for the objective quantification of peripheral nerve asymmetry. We found that the asymmetry measurements (ΔCSA and ΔUtpt) did not significantly differ between the PB and MB patients; moreover, our data indicate a weak tendency toward greater asymmetry in the latter group. The ΔCSA means were greater in MB at the Ut, Upt, and median nerves, and the highest percentages of abnormal ΔCSA values for all studied nerves were found in the LL and BL patients. ΔUtpt showed the same trend, with greater values in the MB patients and the highest percentage of asymmetry in LL patients. We compared only the two polar forms of the disease (TT and LL), aiming to emphasize the findings. Our results confirmed previous analyses, showing significantly greater values of CSA (Upt, Ut, and median nerves) in LL patients. Although asymmetry measurements did not differ significantly between TT and LL patients, we observed a tendency toward greater asymmetry in LL patients. The results for the common fibular nerve showed an opposite tendency, with greater values of ΔCSA (although without statistical significance) in TT patients; nevertheless, we emphasize that one TT patient had the maximum ΔCSA value at common fibular nerve (52.7mm^2^), which could have increased the mean of this group.

Leprosy reactions, especially acute neuritis, can lead to severe nerve impairment and require immediate treatment with steroids and other immunosuppressive drugs. Consistent with the results of previous studies, our results indicate that more severe enlargement and asymmetry of nerves occurs in MB patients with previous or active reactions in all evaluated nerves except for the common fibular nerve. The nerve measurements among PB patients with and without reactions did not show significant differences. Despite the fact that all patients included in our study were examined before beginning WHO multi-drug treatment, the majority of patients who had a history of reactions were already receiving anti-reaction treatment (prednisone, thalidomide and/or azathioprine) at the time of the US exam; thus, nerve swelling and asymmetry might have been diminished in these patients. Taken together with previous results, these findings indicate that chronic *M*. *leprae* nerve infection and its ability to cause inflammation and fibrosis, as well as the presence of leprosy reactions, are important causes of nerve thickening and asymmetry. Previous studies have addressed the influence of reactions on peripheral nerve imaging findings. Martinoli et al. [10] investigated US and magnetic resonance imaging findings for 23 leprosy patients and concluded that patients with previous or active leprosy reactions had nerve enlargement and fascicular abnormalities. These authors also identified the presence of intraneural color Doppler signal in patients with active reactions. Jain et al. [11] have found that increased blood flow can be present in multiple nerves distant to the affected dermal lesion. In our study, we investigated all patients for the presence of Doppler signal; however, because most of the patients with clinical signs of reactions were already receiving anti-reaction treatment at the time of the US exam, Doppler signals were observed only in a small number of them, and the Doppler results are not reported here.

One limitation of our study is the lack of neurophysiological correlation, which could provide useful information concerning nerve function abnormalities. However, our main objective was the evaluation of anatomic alterations, represented by nerve thickening and asymmetry. Two other studies in which ulnar nerve neuropathy was investigated using US and electrophysiology [8,16] found US abnormalities in patients with normal neurophysiological findings. Those studies also demonstrated that leprosy patients can have normal ulnar nerve US findings with significant electrophysiological changes. The authors concluded that leprosy patients can exhibit abnormal nerve anatomy with preserved nerve function and vice versa [8]. Therefore, although we did not perform electrophysiological tests, we consider that our results improve the understanding of anatomic changes in leprosy neuropathy.

Despite the fact that the sample size of this study is the largest reported for any study of the use of US for leprosy neuropathy evaluation, the division of the patients into six clinical classification types resulted in the presence of a small number of subjects in each group. Perhaps the numbers of patients included in the groups with the polar forms TT and LL were not large enough to reveal significant differences in asymmetry measurements between the groups.

In conclusion, the present study contributes to the understanding of the pattern of peripheral nerve involvement in leprosy patients. The US results reported here have revealed that thickening and asymmetry are common in leprosy patients and that these abnormalities occur at similar frequencies in PB and MB. Moreover, the magnitude of thickening was greater in the MB patients and in those with previous leprosy reactions. Nerve asymmetry did not significantly differ between the PB and MB patients, demonstrating that asymmetry is a characteristic of leprosy neuropathy regardless of its classification.

## Supporting Information

S1 Table Clinical data and CSA measurements of each patient included in the study. ID: patient identification; RJ: Ridley-Jopling classification; WHO: Operational classification proposed by the World Health Organization; Upt: ulnar nerve, proximal to the cubital tunnel; Ut: ulnar nerve at the cubital tunnel; TT: tuberculoid; BT: borderline-tuberculoid; BB: borderline-borderline; BL: borderline-lepromatous; LL: lepromatous; PB: paucibacillary; MB: multibacillary; NP: measurement not performed (amputation, cutaneous ulcers or other cutaneous alterations at the site of examination).
(DOCX)
