# Supplementary material for: Asymmetric Nerve Enlargement: A Characteristic of Leprosy Neuropathy Demonstrated by Ultrasonography
Source: PLoS Negl Trop Dis. 2015 Dec 8;9(12):e0004276. doi: 10.1371/journal.pntd.0004276 (PMC4672904; doi:10.1371/journal.pntd.0004276)
Supplement: S1 Table — ID: patient identification; RJ: Ridley-Jopling classification; WHO: Operational classification proposed by the World Health Organization; Upt: ulnar nerve, proximal to the cubital tunnel; Ut: ulnar nerve at the cubital tunnel; TT: tuberculoid; BT: borderline-tuberculoid; BB: borderline-borderline; BL: borderline-lepromatous; LL: lepromatous; PB: paucibacillary; MB: multibacillary; NP: measurement not performed (amputation, cutaneous ulcers or other cutaneous alterations at the site of examination). (DOCX) [file pntd.0004276.s001.docx]

**Table S1. Clinical data and CSA measurements of each patient included in the study.**

|  |  |  |  | CSA measurements (mm²) for each nerve | | | | | | | |
| --- | --- | --- | --- | --- | --- | --- | --- | --- | --- | --- | --- |
| ID | RJ | WHO | Reactions prior to US | Right Upt | Right Ut | Right median | Right common fibular | Left Upt | Left Ut | Left median | Left common fibular |
| 1 | TT | PB | yes | 6 | 7 | 7 | 15 | 6 | 7 | 7 | 13 |
| 2 | TT | PB | no | 6.9 | 6 | 5.3 | 11.2 | 5.2 | 7.2 | 6.8 | 10.6 |
| 3 | TT | PB | no | 10 | 5 | 6 | 5 | 6 | 6 | 7 | 5 |
| 4 | TT | PB | no | 8.1 | 7.3 | 6.5 | 38 | 8.5 | 6 | 6 | 24.6 |
| 5 | TT | PB | yes | 8 | 13 | 8 | 10 | 12 | 7 | 7 | 10 |
| 6 | TT | PB | no | 5.3 | 6.1 | 5.4 | 9.6 | 10.8 | 5.6 | 5.4 | 9.2 |
| 7 | TT | PB | no | 7.5 | 17.4 | 6.5 | 12.5 | 7.1 | 8.2 | 7.4 | 13.1 |
| 8 | TT | PB | no | 7 | 8 | NP | 10.4 | 6.8 | 8.8 | NP | 63.1 |
| 9 | TT | PB | no | 6.5 | 28.5 | 6.7 | 17.9 | 7.4 | 13.6 | 5.2 | 16.9 |
| 10 | TT | PB | yes | 5.3 | 5.2 | 6.5 | 23 | 6 | 6.8 | 5.4 | 30 |
| 11 | TT | PB | no | 3.8 | 4.8 | 4 | 9.3 | 3.5 | 3.9 | 4.4 | 11.3 |
| 12 | BT | MB | no | 7.8 | 6.8 | 8 | 6.8 | 7.5 | 8.9 | 7 | 2.7 |
| 13 | BT | MB | no | 5 | 7 | 6 | 23 | 8 | 6 | 5 | 19 |
| 14 | BT | MB | no | 7 | 8 | 7 | 13 | 7 | 7 | 10 | 16 |
| 15 | BT | MB | no | 7.1 | 6.9 | 5.2 | 10.2 | 6.9 | 7.2 | 5.1 | 10.9 |
| 16 | BT | MB | yes | 6.7 | 15.8 | 8 | 13.4 | NP | 112 | 10.8 | 13 |
| 17 | BT | MB | no | 9.8 | 10.8 | 13 | 18.6 | 8.7 | 11.6 | NP | 15.1 |
| 18 | BT | MB | no | 8.1 | 11.1 | 9.7 | 21.3 | 8.6 | 12.7 | 7.1 | 9.8 |
| 19 | BT | MB | no | 6.1 | 11 | 6.8 | 11.9 | 8.8 | 10.2 | 5.5 | 10.8 |
| 20 | BT | MB | no | 4.1 | 5.5 | 5.8 | 14 | 4.2 | 5.3 | 5.6 | 13.5 |
| 21 | BT | MB | no | 4.2 | 4.7 | 4.5 | 40 | 4.2 | 7.4 | 5.9 | 19.6 |
| 22 | BT | MB | no | 2.8 | 4 | 2.6 | 2.6 | 3.3 | 4.1 | 2.8 | 2.7 |
| 23 | BT | MB | no | 8 | 9 | 11 | 40 | 7 | 8 | 12 | 50 |
| 24 | BT | MB | no | 5 | 11 | 6 | 24 | 10 | 9 | 10 | 20 |
| 25 | BT | MB | no | NP | 27 | 11 | 12 | NP | 29 | 9 | 14 |
| 26 | BT | MB | no | 12.5 | 8.5 | 7.7 | 20.5 | 10.2 | 7.7 | 6.9 | 27.6 |
| 27 | BT | MB | no | 6 | 21.7 | 6.1 | 16.9 | 6.5 | 25.1 | 5.9 | 27.9 |
| 28 | BT | MB | yes | 8 | 9 | 9 | 15 | 8 | 7 | 11 | 16 |
| 29 | BT | MB | yes | 7.1 | 8.6 | 11.1 | 12.4 | 57 | 15.2 | 10.9 | 11.6 |
| 30 | BT | MB | no | 4.4 | 5.8 | 5.3 | 10.8 | 28.1 | 7 | 4.7 | 9.3 |
| 31 | BT | MB | no | 6.2 | 6.5 | 7 | 19 | 7.7 | 6.1 | 6.1 | 21 |
| 32 | BT | MB | no | 7.2 | NP | 4.2 | 11.9 | 13.6 | 7.7 | 17.6 | 45.2 |
| 33 | BT | MB | yes | 8 | 6 | 6 | 15 | 9 | 6 | 6 | 13 |
| 34 | BT | MB | no | 9.5 | 8.8 | 6.1 | 9.6 | 39.8 | 20.9 | 5.1 | 9 |
| 35 | BT | MB | no | 8 | 31 | 6 | 9 | NP | NP | 10 | 9 |
| 36 | BT | MB | no | 6.8 | 7 | 8 | 14.1 | 5.7 | 5.6 | 6.5 | 15.3 |
| 37 | BT | MB | no | 5 | 7 | 6 | 10 | 5 | 6 | 7 | 9 |
| 38 | BT | MB | yes | 5 | 6 | 6 | 8 | 12 | 26 | 6 | 8 |
| 39 | BT | MB | no | 6 | 9 | 7 | 25 | 7 | 6 | 8 | 25 |
| 40 | BT | MB | no | 6.5 | 23.8 | 27.7 | 27.2 | 8.4 | 30.6 | 27.5 | 15.5 |
| 41 | BT | MB | no | 6 | 7 | 7 | 16 | 7 | 8 | 7 | 16 |
| 42 | BT | MB | no | 6 | 8 | 6 | 13 | 6 | 9 | 7 | 15 |
| 43 | BB | MB | no | 12.8 | 14 | 6.3 | 12.7 | 10.5 | 7.9 | 6.3 | 10.1 |
| 44 | BB | MB | no | 15 | 24 | 8 | 11 | 7 | 8 | 8 | 7 |
| 45 | BB | MB | no | 7 | 12.5 | 6.5 | 14.7 | 6.5 | 15.8 | 9.2 | 15.6 |
| 46 | BB | MB | yes | 9.4 | 14.1 | 17.4 | 19.8 | 17.7 | 10.7 | 23.8 | 27.2 |
| 47 | BB | MB | no | 4.4 | 8.1 | 5.8 | 9.3 | 4.6 | 7.4 | 6.9 | 11 |
| 48 | BB | MB | no | 6.3 | 7.5 | 6.2 | 8.5 | 8.5 | 6.6 | 8.6 | 12.5 |
| 49 | BB | MB | no | 7 | 34 | 8 | 9 | 4 | 7 | 8 | 9 |
| 50 | BB | MB | no | 8 | 9 | NP | NP | 6 | 7 | NP | NP |
| 51 | BB | MB | yes | 15 | 13.8 | 8.7 | 9.8 | 10.2 | 11.4 | 7.8 | 9.5 |
| 52 | BB | MB | no | 8.1 | 7.4 | 7.9 | 12.9 | 8.1 | 7.2 | 7.1 | 11.2 |
| 53 | BB | MB | no | 22 | 23.9 | 9.5 | 12.8 | 6.3 | 7.4 | 7.4 | 10.9 |
| 54 | BB | MB | no | 8.8 | 5.4 | 5.1 | 12.1 | 7.4 | 8.3 | 4.9 | 11.9 |
| 55 | BB | MB | no | 8.4 | 10.9 | 5.4 | 11.9 | 8 | 8.5 | 6.8 | 12.1 |
| 56 | BB | MB | no | 4.7 | 8.9 | 4.7 | 8.2 | 4.2 | 5.1 | 6.2 | 12.2 |
| 57 | BB | MB | no | 16 | 13 | 21 | 28 | 28 | 18 | 9 | 25 |
| 58 | BB | MB | no | 4 | 3.4 | 4.9 | 8.8 | 3.9 | 4.7 | 5.1 | 5.7 |
| 59 | BB | MB | yes | 2.9 | 5.6 | 4.5 | 29.5 | 8.2 | 6.2 | 6.4 | 37 |
| 60 | BB | MB | no | 9.7 | 14.8 | 9.4 | 21.2 | 10.6 | 9.9 | 20 | 21.5 |
| 61 | BB | MB | no | 6 | 8 | 6 | 11 | 8 | 8 | 5 | 12 |
| 62 | BB | MB | no | 10 | 12 | 8 | 45 | 12 | 15 | 9 | 25 |
| 63 | BB | MB | no | 8 | 8 | 9.2 | 12.9 | 6.6 | 8.1 | NP | 18 |
| 64 | BB | MB | no | 5 | 13.1 | 12.4 | 8.2 | 6.5 | 9.1 | 10 | 13 |
| 65 | BB | MB | no | 7 | 8 | 7.2 | 11.5 | 5.1 | 8.1 | 6.8 | 10.7 |
| 66 | BB | MB | no | 5 | 5 | 8 | 15 | 5 | 6 | 8 | 15 |
| 67 | BB | MB | no | 6.7 | 17.6 | 6.5 | 34.9 | 6.9 | 12.3 | 6.8 | 40.9 |
| 68 | BB | MB | yes | 29.5 | 22.4 | 5.6 | 10.8 | 47.9 | 35.7 | 10.2 | 9.6 |
| 69 | BB | MB | no | 5.6 | 6.5 | 7.3 | 15 | 6.7 | 6.8 | 6.9 | 14.2 |
| 70 | BB | MB | no | 8.9 | 12 | 9 | 13 | 7.8 | 13 | 9 | 13 |
| 71 | BB | MB | yes | 6 | 7 | 7 | 17 | 9 | 14 | 25 | 14 |
| 72 | BB | MB | no | 3.7 | 7.5 | 4.2 | 16.8 | 4.9 | 7.8 | 5 | 13.8 |
| 73 | BB | MB | no | 6 | 7 | 6 | 24.2 | 5 | 6 | 6 | 29.5 |
| 74 | BB | MB | no | 9 | 12 | 12 | 23 | 10 | 15 | 13 | 31 |
| 75 | BB | MB | no | 4.8 | 7.7 | 4.9 | 11.3 | 4.1 | 7.8 | 5.3 | 12.9 |
| 76 | BL | MB | yes | 8 | 7.7 | 7.3 | 15.2 | 6.7 | 7.8 | 6.4 | 16.9 |
| 77 | BL | MB | yes | 7.2 | 6.5 | 5.4 | 13.8 | 18.1 | 11.2 | 6.2 | 14 |
| 78 | BL | MB | no | 9.8 | 6.6 | 15.9 | 25.3 | 10.3 | 8.6 | 14.1 | 16.9 |
| 79 | BL | MB | yes | 6.7 | 9.8 | 8.6 | 43 | 7.4 | 11.3 | 7.4 | 32.9 |
| 80 | BL | MB | yes | 4 | 8 | 7 | 11 | 5 | 7 | 5 | 9 |
| 81 | BL | MB | yes | 8.8 | 10.4 | 21.5 | 20.5 | 14.3 | 17 | 20 | 25 |
| 82 | BL | MB | yes | 19.8 | 11.5 | 23.5 | 22.2 | 21.3 | 13.4 | 23.2 | 40.2 |
| 83 | BL | MB | yes | 7.4 | 6 | 8.5 | 27.9 | 6.7 | 7.6 | 6.3 | 21 |
| 84 | BL | MB | yes | 21.5 | 12.8 | 8.5 | 12.1 | 25.2 | 10 | 6.4 | 11.9 |
| 85 | BL | MB | no | 4.8 | 5.8 | 7.7 | 46 | 4.7 | 5.4 | 8.7 | 15 |
| 86 | BL | MB | yes | 11 | 18 | 8 | 16 | 13 | 26 | 8 | 15 |
| 87 | BL | MB | yes | 5.9 | 6.2 | 10.7 | 13 | 11.8 | 12 | 20.1 | 12.1 |
| 88 | BL | MB | yes | 17 | 14 | 19 | 23 | 17 | 14 | 18 | 30 |
| 89 | LL | MB | yes | 15.8 | 18.4 | 8.9 | 16.1 | 28.2 | 20.1 | 7.7 | 16.5 |
| 90 | LL | MB | no | 9.2 | 17.9 | 8.9 | 12 | 6 | 12 | 9 | 11 |
| 91 | LL | MB | no | 8.9 | 10.1 | 8.4 | 19.3 | 9.7 | 8.8 | 8.8 | 20.2 |
| 92 | LL | MB | no | 8.1 | 18.9 | 9 | 14 | 7.7 | 16 | 12.3 | 18.6 |
| 93 | LL | MB | yes | 16 | 10 | 7 | 14 | 40 | 14 | 8 | 16 |
| 94 | LL | MB | yes | 14.8 | 26.3 | 18.6 | 22.1 | 12.2 | 17.7 | 15.8 | 28.1 |
| 95 | LL | MB | no | 19 | 15.4 | 12.5 | 24 | 18.8 | 11.4 | 17.7 | 21.2 |
| 96 | LL | MB | no | 5 | 14 | 20 | 36 | 19 | 18 | 20 | 36 |

Legend: ID: patient identification; RJ: Ridley-Jopling classification; WHO: Operational classification proposed by the World Health Organization; Upt: ulnar nerve proximal to the cubital tunnel; Ut: ulnar nerve at the cubital tunnel; TT: tuberculoid; BT: borderline-tuberculoid; BB: borderline-borderline; BL: borderline-lepromatous; LL: lepromatous; PB: paucibacillary; MB: multibacillary; NP: measurement not performed (amputation, cutaneous ulcers or other cutaneous alterations at the site of examination).
